# Hierarchical transcription factor and regulatory network for drought response in *Betula platyphylla*

**DOI:** 10.1093/hr/uhac040

**Published:** 2022-02-19

**Authors:** Yaqi Jia, Yani Niu, Huimin Zhao, Zhibo Wang, Caiqiu Gao, Chao Wang, Su Chen, Yucheng Wang

**Affiliations:** State Key Laboratory of Tree Genetics and Breeding, Northeast Forestry University, 26 Hexing Road, Harbin 150040, China

## Abstract

Although many genes and biological processes involved in abiotic stress responses have been identified, how they are regulated remains largely unclear. Here, to study the regulatory mechanism of birch (*Betula platyphylla*) responding to drought induced by polyethylene glycol 6000 (20%, w/v), a partial correlation coefficient-based algorithm for constructing a gene regulatory network (GRN) was proposed, and a three-layer hierarchical GRN was constructed, including 68 transcription factors and 252 structural genes. A total of 1448 predicted regulatory relationships are included, and most of them are novel. The reliability of the GRN was verified by chromatin immunoprecipitation (ChIP)–PCR and qRT–PCR based on transient transformation. About 55% of genes in the bottom layer of the GRN could confer drought tolerance. We selected two TFs, *BpMADS11* and *BpNAC090*, from the top layer and characterized their function in drought tolerance. Overexpression of *BpMADS11* and *BpNAC090* reduces electrolyte leakage, reactive oxygen species (ROS) and malondialdehyde (MDA) contents, giving greater drought tolerance than wild-type birch. According to this GRN, the important biological processes involved in drought were identified, including ‘signaling hormone pathways’, ‘water transport’, ‘regulation of stomatal movement’, and ‘response to oxidative stress’. This work indicated that *BpERF017*, *BpAGL61*, and *BpNAC090* are the key upstream regulators of birch drought tolerance. Our data clearly revealed that upstream regulators and transcription factor–DNA interaction regulate different biological processes to adapt to drought stress.

## Teaser

A method for construction and verification of gene regulatory networks is proposed. Using this method, a gene regulatory network of birch was constructed and verified, and the key regulators and gene interactions involved in drought response were identified.

## Introduction

*Betula platyphylla* (birch) is a popular landscape tree that is widely distributed in Eurasia. Birch trees grow well in full sun or partial shade, and feature a rounded shape, beautiful silvery bark, fast growth, and medium size, making them capable of providing much beauty in gardens. The many species and varieties of birch include ‘Snow Queen’ (*Betula utilis*), river birch (*B. nigra*), cherry birch (*B. lenta*), and canoe birch (*B. papyrifera*), endowing the bark of birch trees with more color and texture, and a haunting beauty. Birch trees have a generally upright nature, making them a great tree choice for gardeners who do not have enough space to grow larger, spreading trees.

Drought stress is a commonly encountered adverse environmental feature and an important factor that affects the growth of garden plants. In addition, it is estimated that drylands will cover 50% of the land area at the end of this century [1]. Now, about 38% of the world population are living in drylands [1, 2]. To cope with and adapt to a drought environment, plants have evolved a series of strategies at molecular, physiological, and developmental levels [3]. Many genes or biological processes involved in drought stress have been identified. For instance, *PuC3H35* from *Populus ussuriensis* has been found to confer drought tolerance through improving lignin and proanthocyanidin biosynthesis [4]. The gene regulator from apple tree, *MdBBX7*, confers drought tolerance in apple. In addition, *MIEL1* could mediate degradation of *MdBBX7*, and acts as a negative upstream regulator of *MdBBX7* [5]. The VQ motif-containing protein, *MdVQ37*, was identified, and apple plants overexpressing *MdVQ37* displayed reduced drought tolerance. Further studies showed that *MdVQ37* could alter leaf anatomy and salicylic acid homeostasis, leading to sensitivity to drought stress [6]. An R2R3-type MYB transcription factor (TF) from yellowhorn, *XsMYB44*, was upregulated by drought stress. Plants with knock-down of *XsMYB44* displayed reduced drought and heat stress tolerance accompanied with increased reactive oxygen species (ROS) levels and reduced antioxidant enzyme activities [7].

Although numerous drought-related genes were identified, the regulatory networks responding to abiotic stress are still limited; these are regulated by a series of genes, and these genes comprise a hierarchical gene regulatory network (GRN). A GRN is constituted by some regulators such as transcription factors (TFs) at top and middle levels, and structural genes in the bottom layer. High hierarchical regulators are global modulators responding to various cellular signals and environmental cues. The middle-level genes play roles in passing commands from the regulators in the upper layers down to the terminal genes at the bottom layer for execution [8]. Therefore, building an accurate GRN is critical for revealing the genetic regulation process, and is also important for gaining insight into cell functions and biological processes [9].

Recently, a few methods have been developed to establish the regulatory relationships, including TGMI (triple-gene mutual interaction) [10], Bottom-up GGM (Bottom-up Graphical Gaussian Model) [11], ARACNE (Algorithm for the Reconstruction of Accurate Cellular Networks) [12], and BWERF (Backward Elimination Random Forest) algorithms [13]. GRNs have been constructed to reveal the molecular mechanisms of different biological processes. For instance, to investigate the phosphate starvation response, Shi *et al*. [14] constructed a regulatory network between mycorrhizal symbiosis-related genes and TFs using the yeast one-hybrid method. This GRN centered on the TFs in response to phosphate starvation and governed by the conserved P-sensing pathway. Vermeirssen *et al*. [15] presented a GRN based on a microarray array of *Arabidopsis thaliana*. According to this GRN, a regulatory network involved in intricate core oxidative stress was identified, in which the TFs *ERF6*, *WRKY6*, *NAC13*, *NAC032*, and *NAC053* interconnect and function in detoxification. A global *Fusarium graminearum* GRN was constructed from a collection of transcriptomic data, which revealed the connectivity between regulatory genes and their target genes. In this GRN, some unique functions were enriched, such as DNA replication, cell cycle, translation, transcription, and stress responses [16]. A four-layer transcriptional regulatory network was constructed in poplar, which was directed by *PtrSND1-B1*, a key TF involved in secondary wall formation. This GRN contained 57 interactions between TFs and their targets, in which 17 TFs regulated 27 cell wall biosynthetic genes, suggesting that formation of wood involves regulatory homeostasis governed by TF–DNA and TF–TF combined regulation [17]. An integrated microRNA (miRNA) network, including 66 TFs, 318 miRNAs, and 1712 downstream genes, was constructed for *Arabidopsis*, and the analyses revealed how the hub miRNAs perform multiple regulatory roles and elucidated individual functions of the selected miRNA-containing feed-forward loops [18]. Chen *et al*. [19] sequenced the genome of *B. platyphylla* and built a GRN that linked six mitogen-activated protein kinases and presented the MEKK1-MKK2-MPK4 cascade in cold tolerance of birch.

The response to drought in plants is tightly controlled by gene regulation, involving the transmission of the stress signal, the induction of genes perceiving drought stress, and the activation of regulatory genes. Therefore, building a GRN is critical for revealing the mechanisms of drought stress resistance. Although drought stress has been studied intensively at the molecular level, and many TFs involved in drought tolerance have been identified and characterized, there is still a lack of a systematic understanding of the GRN orchestrating abiotic stress responses via fine-tuned regulation [15]. Although methods for GRN construction have been proposed, still very few GRNs have been built. Especially, there is a lack of GRNs responding to drought stress. In response to drought stress, birch should have a multiple hierarchical GRN, and some TFs in the top level of the GRN will play a key regulatory role in drought stress and be valuable for identification.

In the present study, we propose a partial correlation algorithm for the construction of GRNs. To reveal the gene expression regulatory network, differentially expressed genes (DEGs) in response to drought stress for 2, 4, 6 and 9 hours were obtained. Based on the expression profiles of these DEGs, a three-layer hierarchical GRN was constructed using a partial correlation algorithm. We also provided a method for verification of the reliability of the GRNs. This GRN provides necessary data for revealing the drought tolerance of birch.

## Results

### Physiological status of birch after drought stress

Birch plantlets were treated with polyethylene glycol (PEG) 6000 for 2, 4, 6 and 9 hours, respectively. No obvious phenotypic differences were observed among the studied plants exposed to drought for 2, 4 and 6 hours. However, after drought stress for 9 hours, the birch plants were slightly wilted (Supplementary Figure S1a). Physiological analysis showed that drought stress led to significantly increased electrolyte leakage rates and malondialdehyde (MDA) contents (Supplementary Figure S1b and c). The chlorophyll contents were significantly reduced after drought stress for 6 and 9 hours (Supplementary Figure S1d). Superoxide dismutase (SOD) and peroxidase (POD) activities increased after drought stress for 2 or 4 hours, respectively, and increased continually during the studied period (Supplementary Figure S1e, f). These results indicated that drought stress triggered physiological responses characteristic of abiotic stress in the studied plants.

### Description of the construction of the gene regulatory network under drought stress

**Figure 1:**
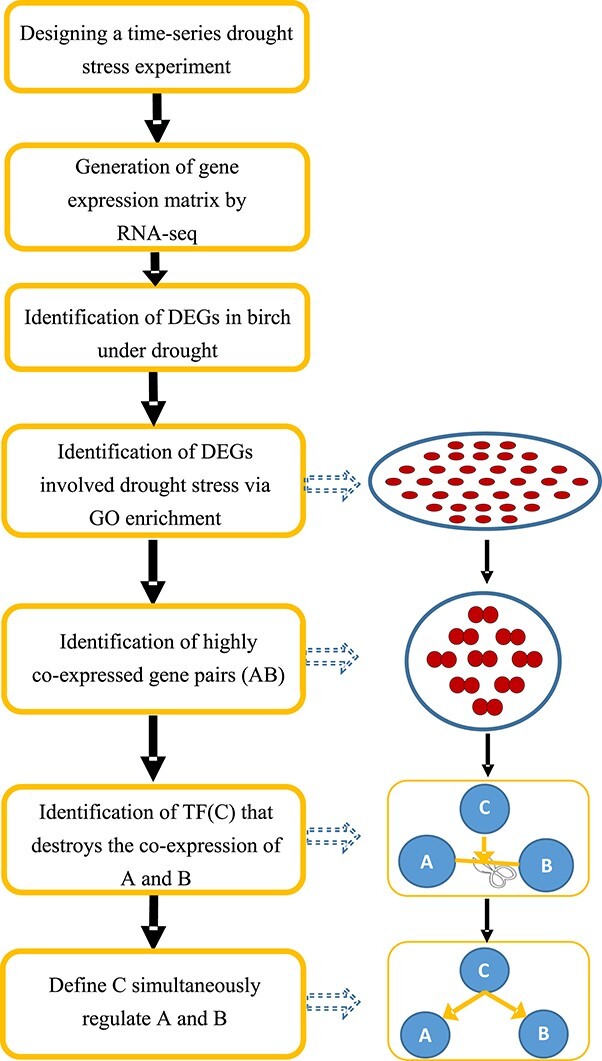
Procedure for the construction of the GRN. The flowchart depicts the steps for constructing the three-layer GRN. First, a stress experiment is designed with a time course for sample harvesting. Then, a gene expression matrix with these samples is generated using RNA-seq. Next, DEGs are identified. Then GO classification is performed to identify the DEGs involved in drought tolerance. Next, a gene pair (termed A and B) with the same expression pattern (co-expression) in these GO terms is determined by calculation of the CCs (CC ≥ 0.8). Next, the TF (termed C) is identified that will prevent the co-expression of A and B when it is introduced (PCC < 0.3), and TF C is assumed to regulate A and B simultaneously. Using this algorithm, a GRN is constructed.

The procedure for constructing a GRN in birch under drought stress is described in Fig. 1. We designed and performed a time-series drought stress experiment and obtained the gene expression matrix using RNA-seq. Under the drought stress condition, thousands of genes in plants were differentially expressed and numerous biological processes have been identified. The GRN controlling drought stress in plants is complicated and difficult to reconstruct. It is nearly impossible to obtain all the regulatory relationships within the DEGs. In this study, we constructed an artificial hierarchical GRN to reveal the potential regulatory relationships among the DEGs. We used biological processes participating in drought stress as ‘baits’ and used a partial correlation algorithm to identify regulators that govern these biological processes. Since the regulatory relationships increase exponentially with the number of DEGs, construction of the GRN was focused on genes involved in biological processes, including ‘jasmonic acid (JA) biosynthetic’, ‘jasmonic acid metabolic process’, ‘response to oxidative stress’, ‘response to jasmonic acid’, ‘response to water deprivation’, ‘response to abscisic acid’, ‘water transport’, ‘response to ethylene’, ‘response to osmotic stress’ and ‘regulation of stomatal movement’. All these biological processes are involved in drought stress according to previous studies.

**Figure 2:**
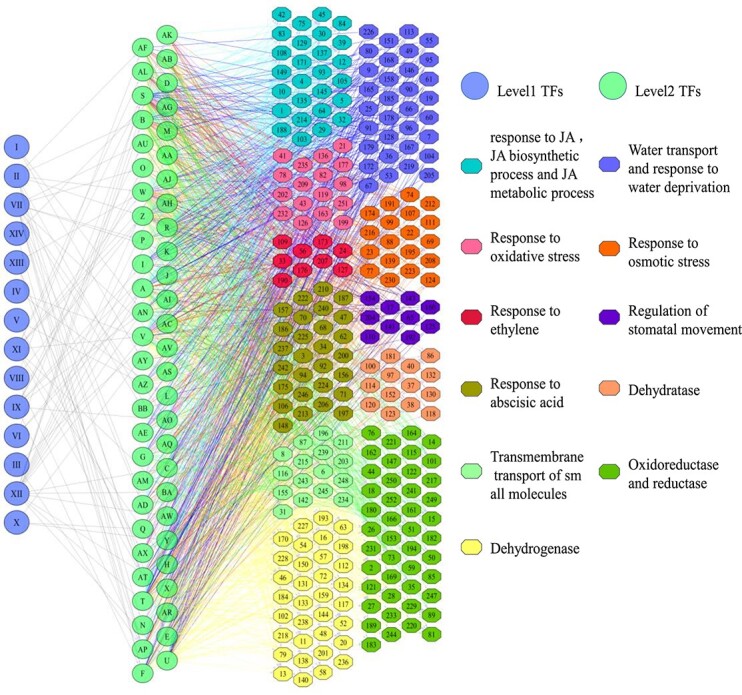
The GRN of birch responding to PEG-induced drought stress. A three-layer GRN was constructed. The first layer contains 14 TFs numbered I–XIV corresponding to *BpERF071*, *BpNAC090*, *BpbZIP53*, *BpZAT10*, *BpWRKY41*, *BpLOB11*, *BpERF2*, *BpERF110*, *BpMADS11*, *BpWRKY11*, *BpAGL61*, *BpERF017*, *BpWRKY6*, and *BpERF113*. The second layer contains 54 TFs, and the bottom layer contains 252 structural genes that are involved in 10 enriched GO biological processes involved in drought tolerance. This GRN was constructed in a drought stress time-series experiment with four time points: 2, 4, 6, and 9 hours. The gene names represented by the numbers in the GRN are shown in Supplementary Data Table S4.

The GRN was then constructed using the prefiltered DEGs from the bottom up. We divided the DEGs into two groups: (i) regulatory genes such as TF-encoding genes, and (ii) structural genes such as enzyme-encoding genes. The structural genes were used as a bottom layer during the GRN construction. Since the expression of genes in the same biological process might be regulated by the same regulator (TF) [20], we calculated correlation coefficients (CCs) for each pair of genes in the bottom layer. Co-expressed gene pairs (A and B) with CCs ≥0.8 were considered to be regulated by the same TF. Next, we calculated partial correlation coefficients (PCCs) for these co-expressed gene pairs by introducing each TF. If TF C significantly destroyed the co-expression relationship between A and B (PCC < 0.3), in other words, A and B are not co-expressed without the interference of C, we assume that TF C regulates A and B simultaneously. Using such an algorithm, we identified a layer of TFs (level 2 TFs in Fig. 2) that may directly regulate the bottom genes (Fig. 2). We then used the layer 2 TFs as a new bottom layer to identify the top layer of TFs (layer 1 TFs in Fig. 2). Finally, a GRN containing three layers was constructed.

### Dynamic changes of birch transcriptome under drought stress

A time series of cDNA libraries, including five time points, was constructed to explore the transcriptional changes of birch under PEG-simulated drought stress. Three independent biological replicates for each time point were generated and a total of 15 cDNA libraries were constructed and sequenced, yielding 25.22–31.68 Mb of reads for each library. Of the reads, 79.40–85.98% were mapped to the birch genome. The DEGs between samples subjected to drought stress and the control were identified. In total, 6291, 6843, 4186, and 5639 DEGs were identified in birch after drought stress for 2, 4, 6, and 9 hours, respectively (Supplementary Data Table S1). Among them, 1786, 1306, 251, and 1060 DEGs were specific to 2, 4, 6 and 9 hours, respectively. By contrast, 1799 genes were differentially expressed at all the studied time points (Supplementary Figure S2a). Most genes were highly altered after stress for 2 and 4 hours, especially after drought stress for 4 hours (Supplementary Figure S2b).

Gene ontology (GO) enrichment analysis was performed to identify DEGs involved in ‘drought stress responsive biological processes’. Enriched GO terms including ‘jasmonic acid (JA) biosynthetic process’, ‘jasmonic acid metabolic process’, ‘response to oxidative stress’, ‘water transport’, ‘response to jasmonic acid’, ‘response to water deprivation’, ‘regulation of stomatal movement’, ‘response to abscisic acid’, ‘response to ethylene’, and ‘response to osmotic stress’ were identified as drought stress-related biological processes (Supplementary Data Table S2). These genes were used for GRN construction.

### Construction of a gene regulatory network in response to drought stress

To further reveal the regulatory relationships among these DEGs, we constructed a GRN using the algorithm described above. A three-layer GRN was constructed, including 14 TFs in the first layer serving as the upstream regulators, 54 TFs in the second layer, and 252 structural genes in the third layer. The 252 genes associated with the 10 GO terms are shown in Supplementary Data Table S3. In total, 1448 potential regulatory relationships between TFs (first layer) and TFs (second layer), and between TFs (second layer) and structural genes (third layer) were identified. The GRN indicates that the expression of the 14 TFs in the first layer was triggered by drought stress, and these TFs serve as global modulators responding to environmental cues to regulate the TFs in the second layer. The commands from the regulators in the first-layer regulators were transferred to the second-layer TFs, and passed down to the genes in the third layer. The genes in the third layer execute specific functions to deal with drought stress (Fig. 2). The gene names represented by the numbers in the GRN are shown in Supplementary Data Table S4.

### Validation of the gene regulatory relationship in the GRN using chromatin immunoprecipitation–PCR and quantitative real-time reverse transcription–PCR

**Figure 3:**
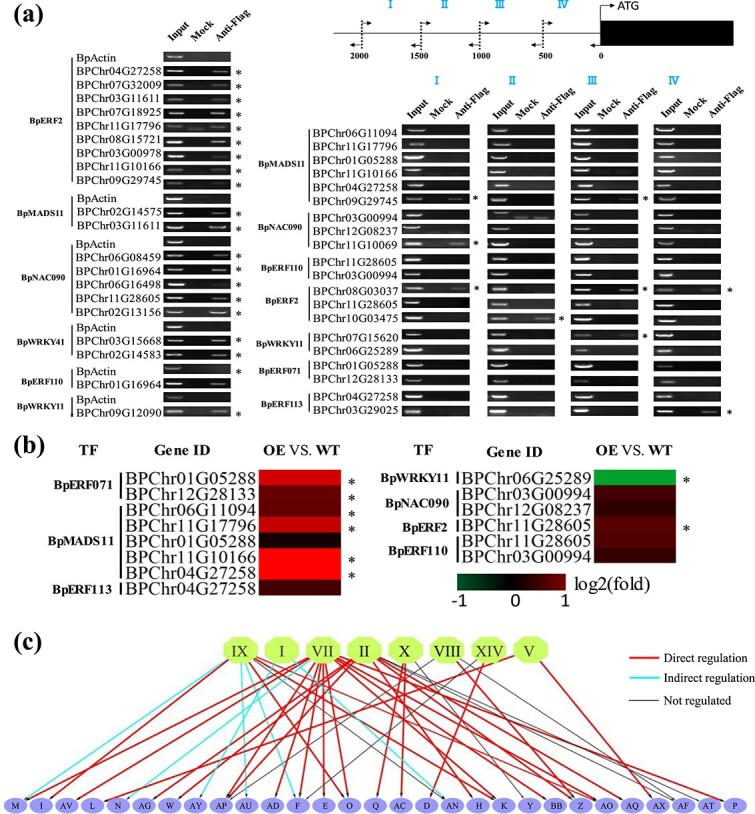
Verification of the regulatory relationship between the first and second layers in the GRN. **a** Verification of the direct regulatory relationships between the TFs in the first and second layers using ChIP–PCR. The binding regions of TFs were predicted first and were amplified by ChIP–PCR (left panel). If the predicted regions failed in ChIP–PCR amplification, the promoters (−2000 to 0 bp) were divided into four equal-length fragments for ChIP–PCR (right panel). Input, mock, and anti-Flag indicate the chromatin before immunoprecipitation (IP), IP with no antibody, and IP with anti-FLAG antibody, respectively. An asterisk (^*^) indicates the binding of the TF to the truncated promoters. **b** Verification of indirect regulatory relationship using qRT–PCR. The heat map displays the fold change (relative expression) of the genes in transiently transformed birch plants overexpressing each TF relative to the control. Red indicates upregulation; green indicates downregulation. An asterisk (^*^) represents a significant regulation relationship (*P* < .05). **c** Summary of the regulatory relationship between the first and second layers in the GRN.

**Figure 4:**
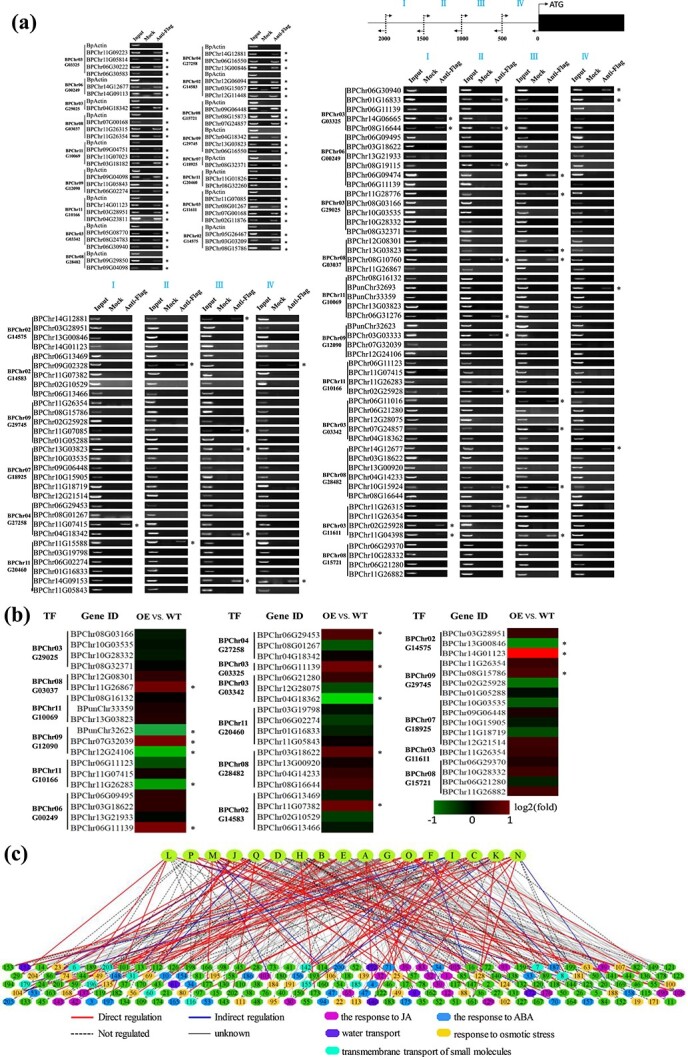
Verification of the regulatory relationship between the second and third layers in the GRN. **a** Verification of the regulatory relationships between the second and third layers using ChIP–PCR. The binding regions of TFs were predicted first and were amplified by ChIP–PCR (left panel). If the predicted regions failed in ChIP–PCR amplification, the promoters (−2000 to 0 bp) were divided into four equal-length fragments for ChIP–PCR. Input, mock, and anti-Flag indicate the chromatin before immunoprecipitation (IP), IP with no antibody, and IP using anti-FLAG antibodies, respectively. An asterisk (^*^) indicates the binding of a TF to the truncated promoters. **b** Verification of the indirect regulatory relationship between the second and third layers using qRT–PCR. The heat map indicates the fold change (relative expression) of the genes in birch plants overexpressing each TF relative to the control (transformed with empty vector). Red indicates upregulation; green indicates downregulation. An asterisk (^*^) indicates a significant difference in gene expression regulation (*P* < .05). **c** Summary of the regulatory relationship between the second and third layers in the GRN.

To evaluate the accuracy of the constructed GRN, we randomly chose some TFs from the first and second layers for chromatin immunoprecipitation (ChIP)–PCR analysis. The binding sites of the studied TFs were predicted in the promoters of their putative target genes, and ChIP–PCR was used to amplify these regions (Figs 3a and 4a). If the predicted binding regions failed in ChIP–PCR amplification, the promoters of these genes (2000 bp in length) were divided into four equal-length fragments and further PCR-amplified (Figs 3a and 4a).

In the first layer, 8 out of 14 TFs were selected and studied. Forty regulatory relationships between these TFs and their putative targets were selected for verification. Among these 40 interactions, 26 were verified by ChIP–PCR (Fig. 3a; Supplementary Figure S3), indicating that these TFs may directly regulate the predicted targets.

Since 14 out of the 40 interactions were not detected by ChIP–PCR, we used qRT–PCR to investigate whether they have indirect regulatory relationships. The results indicated that 8 of these 14 interactions are indirect, i.e. fold change (relative expression) of gene >2 (*P* < .05) (Fig. 3b). Therefore, 65.0% of the predicted interactions may be direct regulatory relationships, and 20.0% may be indirect. In total, 85% of the predicted regulatory relationships in the GRN were experimentally validated. The relationships between the first and second layers are summarized in Fig. 3c.

In total, 137 predicted interactions between the second-layer TFs and their putative targets were selected for verification. According to the ChIP–PCR and ChIP–qPCR results, 83 out of 137 predicted regulatory relationships were confirmed as direct interactions (Fig. 4a; Supplementary Figure S4). The remaining 54 predicted interactions were further studied using qRT–PCR, and 14 of them were identified as indirect regulatory relationships. Among the 137 interactions, 60.5% were confirmed as direct regulations, and 10.0% were indirect regulations (Fig. 4b). Overall, 70.5% of the predicted regulatory relationships between the second and third layers were validated. The direct and indirect regulatory relationships are shown in Fig. 4c.

### Analysis of drought tolerance of structural genes

**Figure 5:**
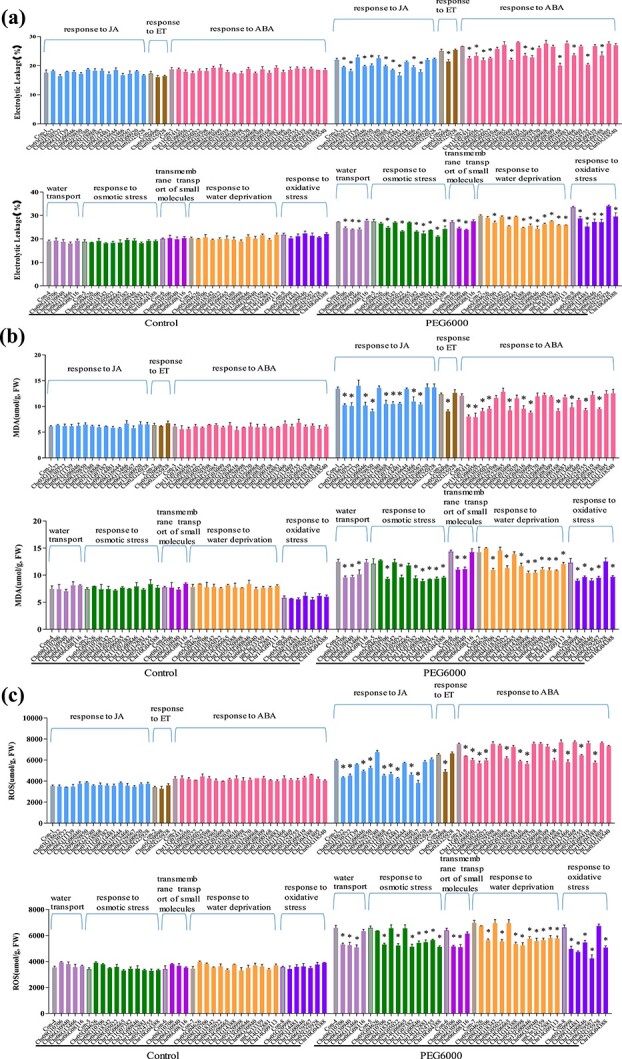
Determination of drought tolerance properties of structural genes in the bottom of the GRN. **a** Electrolyte leakage assay. **b** MDA content analysis. **c** ROS content analysis. The genes were transiently transformed into birch plantlets. At 48 hours after transformation, the plantlets were watered with 20% PEG6000 on roots for 24 hours; transiently transformed plants under normal conditions served as the control. As the transformations were performed in different batches, each batch had an independent control. We selected for study the genes from eight GO processes that are closely involved in drought stress. The GenBank numbers of the genes are shown in Supplementary Data Table S6.

In the GRN, 252 genes were associated with drought response according to GO enrichment. We selected 45 genes from different biological processes for drought tolerance assessment. The physiological traits involved in drought tolerance, such as MDA content, electrolyte leakage, and ROS content were determined. The overexpression of 25 genes showed significantly reduced MDA content, electrolyte leakage, and ROS content simultaneously compared with wild-type (WT) plants when exposed to drought conditions (Fig. 5a–c). The above results suggested that these genes could confer drought tolerance. Therefore, ~55% of the structural genes in the bottom layer were involved in drought tolerance.

### Revealing the drought tolerance mechanism of birch using the gene regulatory network

The GRN showed that when exposed to drought stress, TFs such as ERF, NAC, MADS-box, and WRKY, in the first layer were activated, and these then trigger the expression of other TFs to regulate the structural genes involved in different biological processes, including ‘hormone signaling pathways related’ [abscisic acid (ABA), JA, and ethylene], ‘regulation of stomatal movement’, ‘water transport’, ‘response to oxidative stress’, and ‘response to osmotic stress’ (Supplementary Data Table S2). The changes of these biological processes triggered by TFs finally led to improved drought tolerance of birch (Fig. 2).

In addition, the GRN revealed that in the first layer, *BpERF017*, *BpAGL61* (agamous-like MADS-box) and *BpNAC090* are the top three TFs, regulating more structural genes than other TFs. The results implied that these TFs play essential roles in drought tolerance of birch.

### *BpERF017* is a key regulator that regulates all biological processes in the gene regulatory network

Our data showed that *BpERF017* regulates more TFs and structural genes than any other TFs in the first layer. It regulates 16 TFs in the second layers. These 16 TFs have 622 regulatory relationships with the structural genes and regulate 131 genes that are involved in all 10 biological processes in the GRN. *BpERF017* regulates most of the genes involved in ‘jasmonic acid (JA) biosynthetic process’ and ‘JA metabolic process’, and regulates 50% of the genes involved in ‘response to JA’, suggesting that *BpERF017* is closely related to the JA signaling pathway. In addition, *BpERF017* is the main regulator of the processes ‘response to water deprivation’, ‘response to oxidative stress’, ‘response to osmotic stress’, and ‘water transport’, because it regulates >20 genes or 50% of total genes in these processes. Therefore, the results together suggested that *BpERF017* is a key upstream regulator involved in drought response in birch.

### *BpAGL61* (agamous-like MADS-box) is a main regulator in this GRN

*BpAGL61* regulates nine TFs in the second layer, and these nine TFs have 289 regulatory relationships with their downstream genes and regulate 118 structural genes. *BpAGL61* also regulates most genes in ‘JA biosynthetic process’, ‘JA metabolic process’, and ‘response to JA’, suggesting that it is closely related to the JA signaling pathway. At the same time, seven out of nine genes in ‘response to ethylene’ were regulated by *BpAGL61*, indicating that *BpAGL61* is also tightly involved in the ethylene signaling pathway. Furthermore, *BpAGL61* regulates most genes in the process of ‘regulation of stomatal movement’ and ‘response to osmotic stress’. The above results suggested that *BpAGL61* is an important upstream regulator involved in drought response that confers drought tolerance through regulating multiple processes.

### *BpNAC090* is an important regulator in the gene regulatory network

*BpNAC090* regulates eight TFs, which have 159 regulatory relationships with the genes in the third layer and regulate 97 structural genes. Compared with *BpAGL61* and *BpERF017*, *BpNAC090* rarely regulates genes involved in ‘JA biosynthetic process’, ‘JA metabolic process’, and ‘response to JA’, indicating that *BpAGL61* is not involved in the JA signaling pathway. The genes related to ‘water transport’ were also rarely regulated by *BpNAC090*. However, it regulates many genes related to ‘response to water deprivation’, ‘response to oxidative stress’, ‘regulation of stomatal movement’, and ‘response to osmotic stress’, suggesting that *BpNAC090* confers drought tolerance mainly by regulation of these processes.

### Overexpression of *BpNAC090* and *BpMADS11* confers drought tolerance

**Figure 6:**
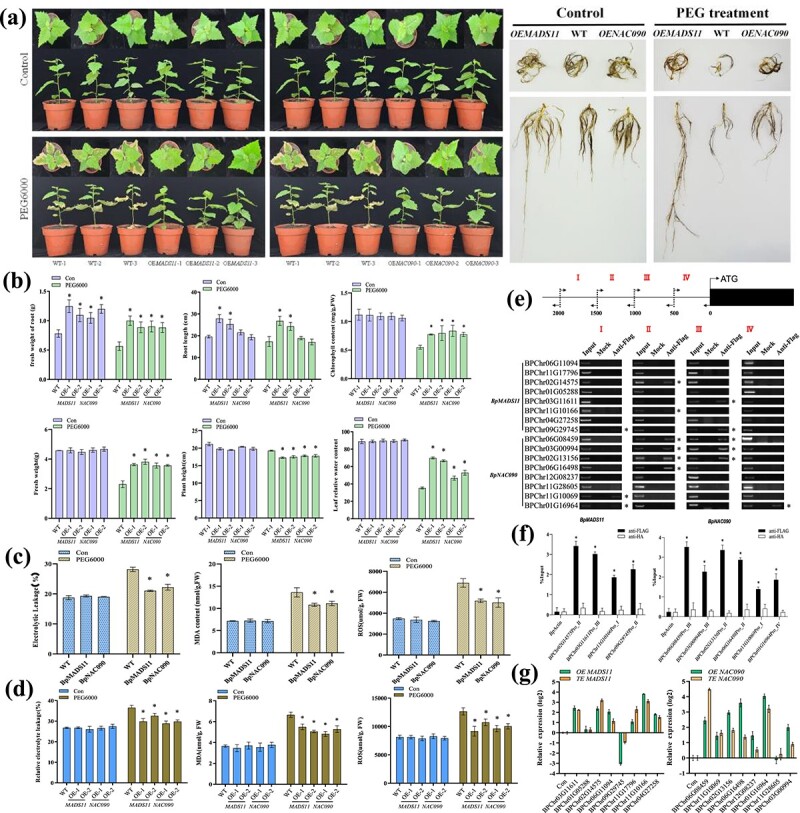
Determination of drought tolerance of birch plants stably overexpressing *BpNAC090* and *BpMADS11*. **a** Comparison of the growth phenotype between WT and transgenic plants overexpressing *BpANAC090* and *BpMADS11*. The birch lines were treated with PEG6000 (9% w/v) for 15 days and their growth phenotypes were compared. **b** Analysis of phenotype traits and relative water contents between transgenic plants and WT plants. An asterisk (^*^) indicates a significant difference in gene expression regulation (*P* < .05). **c** Analysis of MDA, electrolyte leakage, and ROS content in transiently transformed plants overexpressing *BpNAC090* and *BpMADS11*. **d** Analysis of MDA, electrolyte leakage, and ROS content in plants stable overexpressing *BpNAC090* and *BpMADS11*. **e** Assay of the binding of *BpNAC090* and *BpMADS11* to the promoters of their predicted target genes using ChIP–PCR. **f** ChIP–qPCR analysis of the direct interaction identified by ChIP–PCR. Three independent replicates were performed, and the error bars indicate the standard error. An asterisk (^*^) indicates a significant enrichment compared with control (anti-HA). **g** Comparison of gene expression regulated by *BpNAC090* and *BpMADS11* between transiently transformed and stably transformed plants. OE, stably transformed plants overexpressing *BpNAC090* and *BpMADS11*; TE, transiently transformed plants overexpressing *BpNAC090* and *BpMADS11*.

Two TFs encoding genes in the first layer, *BpNAC090* and *BpMADS-box* (*BpMADS11*), were selected for functional characterization. They were stably transformed into birch plants, and the overexpression of the two genes was confirmed by qRT–PCR (Supplementary Figure S5). The transgenic lines with relatively high expression levels of *BpNAC090* and *BpMADS-box* were used for further study (Fig. 6a). After drought stress (10% w/v PEG6000) for 15 days, their growth phenotype was investigated. The results showed that all the studied plant shared similar heights (Fig. 6b). However, the fresh weight of the plants overexpressing *BpNAC090* and *BpMADS11* were both higher than those of the WT plants, either under drought or normal conditions (Fig. 6b). The root length of birch plants overexpressing *BpMADS11* was significantly higher than those of WT plants under both normal and drought treatment conditions (Fig. 6b), indicating that *BpMADS11* plays a role in root growth in addition to drought tolerance. At the same time, the number of lateral roots of birch plants overexpressing *BpNAC090* was significantly higher than that of WT plants when exposed to drought conditions (Fig. 6b). Furthermore, the relative water contents of these studied plants were similar; however, under drought stress conditions, plants overexpressing *BpNAC090* and *BpMADS11* had higher relative water contents than the WT plants (Fig. 6b). These results suggested that *BpNAC090* and *BpMADS11* could confer drought tolerance on transgenic plants, which is consistent with the prediction of the GRN.

To study the reliability of the physiological investigation based on transient transformation, physiological analyses were performed using both stably and transiently transformed plants overexpressing *BpNAC090* and *BpMADS11*. The results showed that the MDA content, electrolyte leakage, and ROS content in the transiently transformed plants overexpressing *BpNAC090* and *BpMADS11* were all significantly reduced in comparison with those in WT plants (Fig. 6c). Consistently, the stably transformed birch plants overexpressing *BpNAC090* and *BpMADS11* also showed reduced electrolyte leakage, MDA content and ROS content in comparison with WT plants (Fig. 6d), suggesting that physiological studies based on transient transformation are reliable.

To further verify the reliability of ChIP results based on transient transformation, ChIP–qPCR and ChIP–PCR were carried out using stably transformed plants expressing *BpNAC090* and *BpMADS11*. The ChIP–PCR and ChIP–qPCR results for *BpNAC090* and *BpMADS11* (Fig. 6e and f) were consistent with those obtained from the transiently transformed plants (Fig. 3a; Supplementary Figure S3), suggesting that the ChIP–PCR and ChIP–qPCR results obtained using transiently transformed plants are reliable. qRT–PCR was then performed using cDNA from the stably and transiently transformed plants overexpressing *BpNAC090* and *BpMADS11*. The results showed that the qRT–PCR results from the stably transformed plants were well consistent with the results obtained from the plants transiently expressing *BpNAC090* or *BpMADS11* (Fig. 6g). Collectively, these results showed that physiological analysis, ChIP–PCR, and qRT–PCR based on transient transformation are reliable.

## Discussion

When exposed to drought stress, plants will regulate a series of genes, thus forming a GRN to adapt to drought stress conditions. Therefore, construction of the GRN in response to drought stress is important to reveal the drought tolerance mechanism. However, there is still little investigation on the construction of GRNs in response to abiotic stress. In addition, although some GRNs have been built, it is difficult to verify them, and only very few studies have verified the accuracy of the GRNs. This is because ChIP–PCR and qRT–PCR are needed to investigate the direct or indirect interaction, and these two methods both need a lot of genetic transformation work. However, the genetic transformation work is time-consuming; a very long time will be needed to complete it, making it is impossible to verify the reliability of GRNs.

In this study, we employed a transient transformation we previously developed to perform ChIP–PCR and qRT–PCR [21], which enables this work to be completed within a short time. In addition, we further performed stable genetic transformation to study the reliability of physiological analysis, ChIP–PCR and qRT–PCR based on transient transformation. The results showed that the results of physiological analysis, ChIP–PCR and qRT–PCR based on transient transformation are consistent with those based on stable transformation (Fig. 6). Therefore, the reliability of GRNs could be verified using ChIP–PCR and qRT–PCR based on a transient transformation method, which could be completed within a short time and with a reduced workload.

The co-expression principle has been widely used to construct GRNs in response to abiotic stress, but it is difficult to infer direct regulation between TFs and their targets because it is nearly impossible to identify causal relationships among genes by using their expressional profiles independently. For a pair of co-expressed genes, it is not possible to define a regulatory relationship between them or determine which of the pair the regulator is. Under stress conditions, plants mobilize many biological processes to survive. These alterations are implemented by regulating enzyme activities. At the transcriptional level, the expression levels of these enzyme-encoding genes are mainly regulated by TFs. In this study, we identified putative structure genes or enzyme-encoding genes from the stress-responsive genes and then used a triple-gene model to identify the regulatory relationships between TFs and their targets [10]. In the triple-gene model, a pair of co-expressed structure genes are supposed to be regulated by the same TF. Such a biological model is useful to eliminate superficial regulatory relationships among co-expressed genes and distinguish the regulatory relationships between TFs and their targets. Although the artificial hierarchical GRN built in this study may lose some regulatory relationships, such as relationships within the same layer, it has an advantage over co-expression GRNs. In addition, the ChIP–PCR and qRT–PCR results indicated that the relationships in the hierarchical GRN were reliable and the hierarchical GRN is instrumental in revealing the mechanism of transcriptional regulation under drought stress.

### Jasmonic acid and abscisic acid signaling processes are included in this gene regulatory network signaling process

JA is a plant signaling molecule playing a central role in resistance to abiotic and biotic stress, and the endogenous JA content increases rapidly after drought treatment, which induces a series of genes to mediate responses against environmental stresses [22, 23]. We found that *BpERF017* and *BpAGL61* regulated more structural genes than other upstream regulators in the GRN (Fig. 2; Supplementary Data Table S3). Additionally, previous studies also showed that the *BpERF017*-homologous gene *ATERF71* (AT2G47520) and the *BpAGL61*-homologous gene *AGL16* (AT3G57230) are both involved in abiotic stress tolerance [24, 25]. Therefore, the results together suggested that these two TFs mediate drought tolerance through the JA signaling pathway.

There were two signaling pathways responding to abiotic stress in plants, i.e. ABA-independent and ABA-dependent signaling. The bZIP TF family was found to have a pivotal role in the regulation of the ABA-dependent response [26]. At the same time, MYB and MADS-box are also involved in the ABA-dependent signaling pathway [25, 27]. There were many TFs in the GRN that were involved in the ABA-dependent signaling pathway, including 2 bZIP, 10 MYB, and 2 MADS-box TFs (Fig. 2), suggesting that the ABA-dependent signaling pathway constitutes an important component of the GRN. Therefore, this GRN has integrated both JA- and ABA-dependent signaling pathways.

### *BpAGL61* and *BpNAC090* are the key regulators in drought tolerance of birch

The GRN showed that >40% of genes in the processes of ‘response to osmotic stress’, ‘response to water deprivation’, and ‘response to oxidative stress’ are regulated by *BpAGL61* (Fig. 2; Supplementary Data Table S3), demonstrating that *BpAGL61* plays a main role in regulating these processes. In addition, most of the genes involved in the JA signaling pathway are regulated by *BpAGL61* (Fig. 2). Furthermore, the GRN showed that *BpAGL61* may regulate all the processes involved in drought stress to some extent via other TFs (Fig. 2; Supplementary Data Table S3). Taken together, the above results indicate that *BpAGL61* plays an important role in drought tolerance, and might be a key regulator in drought tolerance of birch.

In this study, we found that *BpNAC090* is in the first layer of the GRN; at the same time, overexpression of *BpNAC090* displays improved drought tolerance, indicating that it plays a role in the drought response. Consistently, previous studies also indicated that the NAC gene *NAC090* (AT5G22380), which shares high sequence similarity with *BpNAC090*, also can confer salt tolerance [28]. Therefore, *BpNAC090* and its homologous genes are important transcription regulators involved in abiotic stress.

### The stomatal movement process was regulated by some specific TFs to improve drought tolerance

When exposed to water-deficiency stress, plants will close their stomas to reduce the loss of water, and the stomatal movement is regulated by different TFs [29, 30]. Ten genes involved in ‘regulation of stomatal movement’ were included in this GRN (Fig. 2), and most of them confer drought tolerance (Fig. 5). However, our data showed that there were five TFs in the first layer, including *BpERF017*, *BpERF2*, *BpAGL61*, *BpWRKY6*, and *BpNAC090*, which could regulate genes related to ‘regulation of stomatal movement’, indicating that the regulation of this process is specific (Fig. 2; Supplementary Data Table S3).

### The processes of ‘response to osmotic stress’ and ‘response to oxidative stress’ play common roles in drought tolerance of birch

Under abiotic stress conditions, plants will accumulate ROS, and an excess of ROS will induce oxidative stress [31, 32]. The GRN contained 50 and 56 genes involved in ‘response to osmotic stress’ and ‘response to oxidative stress’, respectively, and many of them confer drought tolerance (Fig. 5). The GRN indicated that all the upstream TFs in the first layer can regulate the genes involved in these two processes, suggesting that ‘response to osmotic stress’ and ‘response to oxidative stress’ play common roles in drought tolerance and could be generally employed by the TFs to facilitate drought tolerance.

### Conclusions

We propose a PCC-based algorithm to construct a GRN for birch responding to drought stress. The ChIP–PCR and qRT–PCR results confirmed that the regulatory relationships in the GRN (Fig. 2) are reliable. The results suggested that this algorithm is suitable for building GRNs. According to the constructed GRN, the regulatory relationship among TFs and TFs and among TFs and structural genes responding to drought stress are revealed. The TFs involved in some biological processes related to drought tolerance, such as ‘JA signaling, stomatal movement’, ‘response to osmotic stress’, and ‘response to oxidative stress’ were found to play essential roles in adaption to drought stress. The identified regulatory relationships and drought tolerance genes are valuable for molecular breeding for drought tolerance. The GRN developed here also could be exploited to comprehend other abiotic stresses, such as salt and cold, in plants.

## Materials and methods

### Plant materials and drought treatments

Birch plantlets were grown in a growth chamber under the controlled conditions of relative humidity of 70%, temperature of 24°C, and a light/dark cycle of 16/8 hours. Three-month-old birch plantlets were grown in pots containing soil and were well watered. The plants were then watered with 20% (w/v) PEG6000 (osmotic potential ~−0.49 MPa at a temperature of 24°C) [33] solution on their roots for 2, 4, 6, and 9 hours, and plants without PEG treatment were used as a control. All the samples were treated at different times to make sure that they could be harvested at the same time. Each sample contained five seedlings, the seedlings were grown in soil in pots, and one seedling per pot was grown. The leaves of seedlings were harvested, pooled in each sample, and served for physiological analysis and RNA-seq.

### Construction of cDNA library for RNA sequencing

The cDNA libraries were built using the NEBNext Ultra™ RNA Library Prep Kit for Illumina (NEB, Ipswich, MA, USA) following its protocol for RNA-seq, and the Illumina Hiseq 4000 platform (Illumina, San Diego, USA) was used to sequence the libraries.

The RNA-seq data were analyzed by using the RSEM (RNA-Seq by Expectation–Maximization) pipeline according to Li and Dewey [34]. The low-quality reads were discarded, and all the clean reads were aligned to the *B. platyphylla* genome [19] using Bowtie 2 with default parameters [35]. DEGs between each comparison of treatments and control were determined by using the program edgeR, with a threshold false discovery rate of <.05 [36]. The trimmed mean of the M-values method was used to normalize the transcript levels of genes. The minimum value of the count per million was set as 1 to filter out low-expression genes.

### Identification of biological processes involved in drought stress

Birch is a non-model tree species with limited GO annotation; therefore, we annotated the birch genome using *Arabidopsis* annotations. The best hits of birch genes in *Arabidopsis* were identified using the BLASTP program with a threshold E-value of <1E [5]. GO enrichment of the DEGs was then performed to identify overrepresented biological processes in birch under drought stress.

### Construction of a multilayered hierarchical gene regulatory network using a partial correlation coefficient-based algorithm

A PCC-based algorithm was used for the reconstruction of the TF-based GRN (Fig. 1). In brief, the algorithm was based on the fact that co-expressed genes are potentially regulated by the same regulator, such as the same TF. This concept was used to identify the regulatory relationship between TFs and structural genes. We first identified the co-expressed structure genes with thresholds of CC ≥.8 and *P*-value <.001 [37]. These co-expressed genes may be regulated by the same TF. We then calculated the PCC for the co-expressed gene pairs using the algorithm ${r}_{xy\mid z}=\frac{r_{xy}-{r}_{xz}{r}_{yz}}{\sqrt{1-{r}_{xz}^2}\sqrt{1-{r}_{yz}^2}}$. Here *x* and *y* indicate the co-expressed gene pair and *z* indicates a TF. If the PCC of the co-expressed gene pair is <.3 [38], we define the gene pair as regulated by the same TF.

### Plant expression vector construction and transient transformation

For plant overexpression vector construction, the coding sequences of the genes were cloned using RT–PCR, and their sequences were confirmed by Sanger sequencing. The coding sequence of each studied gene was cloned into the *P1307-3xFLAG* vector controlled by the 35S CaMV promoter. The primers for constructing these vectors are shown in Supplementary Data Table S5. Transient transformation was conducted following the method described by Zang *et al*. [39]. In brief, colonies of *Agrobacterium tumefaciens* EHA105 harboring different constructs were each grown in LB medium to an OD_600_ of 0.6–0.7 at 180 rpm at 28°C. The cultures were centrifuged at 3000 × *g*, and the cells were suspended in a transformation solution [1/2 MS (Murashige and Skoog medium) + 120 μM acetosyringone + 2.5% (w/v) sucrose + Tween 20 (0.01%, v/v), pH 5.8] to an OD_600_ of .8. For transient transformation, the plants were soaked in transformation solution for 2 hours with shaking at 90 rpm at 25°C, then washed quickly with distilled water to remove excess *A. tumefaciens* cells. The transformed plants were vertically planted on a solid medium [1/2 MS + 120 μM acetosyringone + 1% (w/v) sucrose, pH 5.8], and cultured for 48 hours and were then used for subsequent treatment and experiments. The GenBank numbers of the genes studied are included in Supplementary Data Table S6.

### Generation of birch plants with stable genetic transformation

The coding sequences of *BpMADS-box* (*BpMADS11*) and *BpNAC090* were fused in-frame with 3 × FLAG controlled with a 35S CaMV promoter, separately, to generate the constructs. The primers for constructing the vectors are shown in Supplementary Data Table S5. The explants of birch were soaked in a suspension of *Agrobacterium* at OD600 of 0.3 for 5 min, and then these explants were cultured on a co-culture medium [Woody Plant Medium (WPM) + 120 μM acetosyringone + 1 mg l^−1^ 6-benzylaminopurine (6-BA) + 3% sucrose, pH 5.6] for 3 days. After co-culture, they were transferred onto a medium to select antibiotic callus (WPM + 300 mg l^−1^ carbenicillin + 50 mg l^−1^ kanamycin + 1 mg l^−1^ 6-BA, pH 5.8). After selection for 4–5 weeks, antibiotic-resistant calluses appeared, which were moved to a medium (WPM + 50 mg l^−1^ kanamycin + 1 mg l^−1^ 6-BA, pH 5.8) for bud generation. Then, the small plants were transferred to a rooting medium (WPM + 50 mg l^−1^ kanamycin + 0.2 mg l^−1^ 1-naphthaleneacetic acid, pH 5.8) to generate roots.

### Drought stress treatment

*BpMADS-box* (*BpMADS11*) and *BpNAC090* transgenic and WT plants were grown in soil and treated with drought stress. For imitation of drought stress, the plants were watered with 10% (w/v) PEG6000 solution on roots for 15 days by applying 150 ml of 10% PEG6000 solution to each flowerpot every 3 days. Then we observed the phenotype and root length, fresh weight, and chlorophyll content and determined physiological indicators. Normally growing plants were used as controls. Each sample contained five plants.

### Chromatin immunoprecipitation (ChIP), ChIP–PCR and ChIP–qPCR

TFs from the first and second layers were fused separately with FLAG tags to generate fusion genes driven by the 35S promoter in vector *35S::TF-3 × FLAG* for overexpression. For the ChIP assays, the construct *35S::TF-3 × FLAG* was transiently transferred into birch plants and was used for the ChIP study. ChIP was conducted following the protocol described by Haring *et al*. [40]. Briefly, the chromatin was sonicated on ice, and was then immunoprecipitated with the anti-FLAG antibody (Sigma) (termed ChIP+) or a rabbit anti-hemagglutinin (HA) antibody (Abcam) as a negative control (termed ChIP−). The New PLACE website (https://www.dna.affrc.go.jp/PLACE/?action=newplace) was employed for prediction of the motifs present in the gene promoter. The TF binding sites in the promoter were determined according to the annotation of New PLACE. ChIP–PCR primers were so designed that the binding sites were contained as far as possible in the middle of PCR-amplified regions. The reaction volume for ChIP–PCR contained 1 μl of ChIP product and 0.5 μM of each primer in 20 μl. ChIP–PCR was carried out with the following thermal parameters: 2 min at 94°C; then 35 cycles of 30s at 94°C, 30 s at 57°C, and 30s at 72°C. For ChIP–qPCR, the reaction volume contained SYBR Premix Ex Taq™ (TaKaRa) (10 μl), ChIP product (1 μl), and forward and reverse primers (0.5 μM of each), and the total volume was 20 μl. ChIP–qPCR was conducted on a qTower 2.2 (Analytik Jena AG, Germany) with the temperature profile of 2 min at 94°C, then 40 cycles for 30 s at 94°C, 30 s at 57°C, and 30 s at 72°C. The experiments were conducted with three biological replicates. All the primers are shown in Supplementary Data Table S7.

### Physiological change analysis

The electrolyte leakage rate was determined following the description of Dionisio-Sese and Tobita [41]. In brief, thoroughly rinsed samples were added to ultrapure water and vacuumed for 15 min, and electrical conductivity was determined (termed S1). Then the sample was incubated at 90°C for 20 min, and electrical conductivity was determined again (termed S2). The formula for electrolyte leakage rate calculation was (S1/S2) × 100%. Proline was determined using the sulfosalicylic acid reaction following the description of Bates *et al*. [42]. MDA was measured following the method of Madhava and Sresty [43]. After the sample had been ground into powder, 10% (w/v) thiobarbituric acid was added, and the sample was incubated at 4°C for 12 hours then centrifuged. An equal volume of 0.6% (w/v) thiobarbituric acid was added to the supernatant and the mixture was incubated in boiling water for 15 min. After centrifugation, the absorbances of the supernatants at wavelengths of 532 and 450 nm were determined, and water was used as the background. The formula of MDA (M) = 6.45 × OD_532_–0.56× OD_450_ was used to calculate MDA content. POD activity was determined using the guaiacol method [21]. SOD was determined with a nitroblue tetrazolium method by spectrophotometry following the method of Zang *et al*. [21]. Chlorophyll was measured spectrophotometrically at 663 and 645 nm. The water loss rate was assessed by measuring the detached leaves at different times under dehydration conditions, according to the method of Hsieh *et al*. [44]. ROS were measured by enzyme-linked immunosorbent assay (ELISA) with a kit from Nanjing Senbeijia Bioengineering Institute (Nanjing, China). The GenBank numbers of the genes are included in Supplementary Data Table S6.

### Quantitative real-time reverse transcription–PCR

Total RNA of birch was isolated using a CTAB method, and was reverse-transcribed using oligo(dT) as primer with a PrimeScript™ RT Reagent Kit (Takara, Dalian, China). The cDNA diluted with ultrapure water to 100 μl was used as the qRT–PCR template. The qRT–PCR system contained forward and reverse primers (0.5 μM of each), SYBR Premix Ex Taq™ (10 μl) (Takara), and the cDNA template (2 μl) with a total volume of 20 μl. qRT–PCR was conducted on a qTower 2.2 (Analytik AG, Jena, Germany) with the following thermal profile: 30 s at 94°C; followed by 40 cycles of 12 s at 94°C, 30 s at 58°C and 45 s at72 °C. The tubulin and ubiquitin genes served as the internal controls. The GenBank accession numbers of the genes together with their primers are included in Supplementary Data Table S8. The 2^-ΔΔCT^ method [45] was used to calculate relative transcriptional abundance. Three biological replications were performed.

### Statistical analysis

One-way analysis of variance (ANOVA) was employed for data analysis. The Statistical Package for the Social Sciences (SPSS 22, IBM Corp., Armonk, NY, USA) was used to perform all the statistical analyses, and a *P*-value <.05 was considered to indicate statistical significance.

## Acknowledgements

This work was supported by the National Nonprofit Institute Research Grant of the Chinese Academy of Forestry (grant number CAFYBB2019ZY003), the National Natural Science Foundation of China (No. 31971684), and the Heilongjiang Touyan Innovation Team Program (Tree Genetics and Breeding Innovation Team).

## Author contributions

Y.W. and C.G. conceived and directed the research. Y.J. performed the experiments and the overall data analysis. S.C. constructed the multilayered hierarchical gene regulatory network. Y.N. and H.Z. provided reagents and analysis tools. Z.W. and C.W. provided plants overexpressing *BpNAC090*. Y.J. and Y.W. wrote the manuscript and all authors revised the manuscript. All authors read and approved the final manuscript.

## Data availability

The data used to support the findings of this study are available from the corresponding authors upon reasonable request.

## Conflict of interest

The authors declare no conflicts of interest.

## Supplementary data

Supplementary data is available at *Horticulture Research* online.

## Supplementary Material

Web_Material_uhac040
