# Supplementary material for: Hierarchical transcription factor and regulatory network for drought response in Betula platyphylla
Source: Hortic Res. 2022 Feb 19;9:uhac040. doi: 10.1093/hr/uhac040 (PMC9070641; doi:10.1093/hr/uhac040)
Supplement: Web_Material_uhac040 [file web_material_uhac040.zip › Figure S1-S5.docx]

**Figure S1**


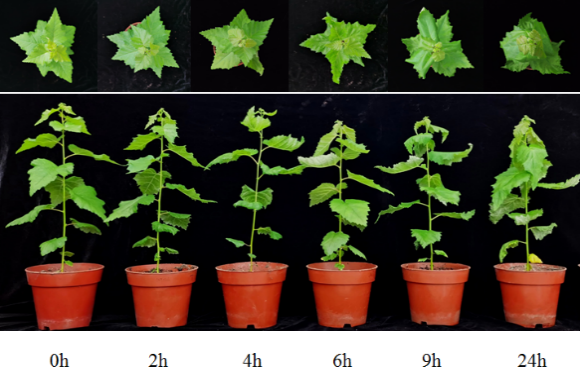


**(a)**


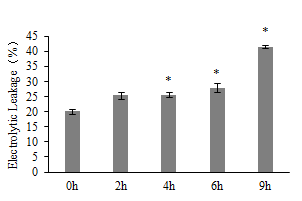


**(b)**

**(c)**

**(d)**


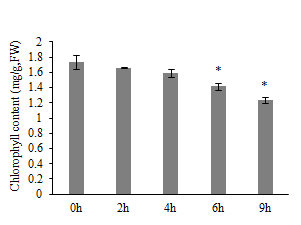


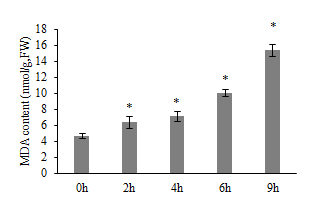


**(f)**

**(e)**


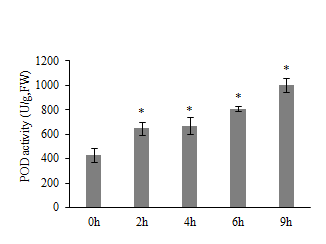

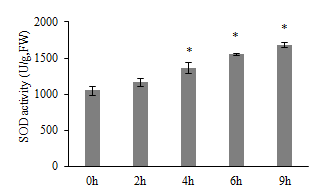


**Figure S1 Determination of the physiological traits of the birch used for RNA-seq analysis.** (a) The growth phenotype of the birch plants used for RNA-seq identification. (b-f) Analysis of the electrolyte leakage (b), MDA content (c), chlorophyll content (d), SOD activity (e), and POD activity (f), under PEG-induced stress at different stress time points. Three-month-old birch seedlings were watered on roots with 20% PEG6000 solution for 0, 2, 4, 6 and 9h, and well watered birch seedlings were used as control.

**Figure S2**

**(a)**

**(b)**


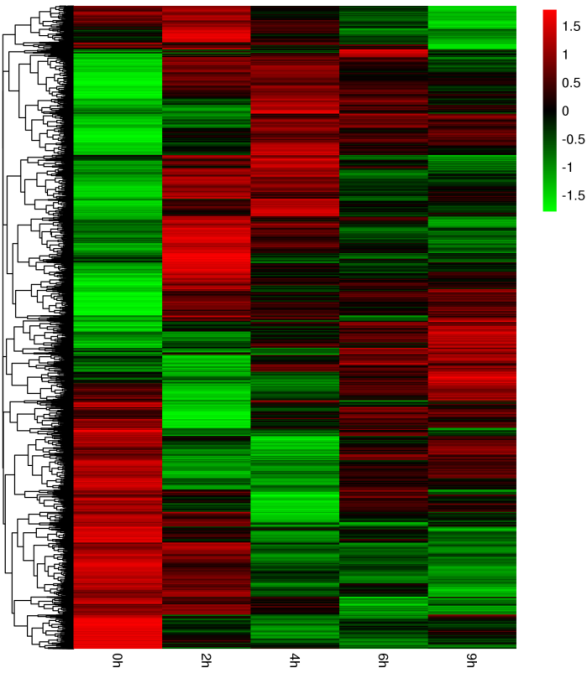

**Figure S2 The characteristics of gene expression of birch at different PEG treatment time points.**

(a) Distribution of the differentially expressed genes under drought stress for 2, 4, 6, and 9 h. (b) Heatmap of gene expression of birch under drought stress for 2, 4, 6, and 9 h.

**Figure S3**

**Figure S3 Determination of the direct regulatory relationship between the up and middle layer of GRN using Chromatin immunoprecipitation (ChIP)-qPCR.** ChIP-qPCR assay of the regulatory relationship among the TFs in the up and second layer. Three independent replicates were performed, and the error bars indicate the standard error. * Indicates a significant enrichment (P<0.05) compared with control (anti-HA).

**Figure S4**

**Figure S4 Determination of the direct regulatory relationship between the middle and bottom layer of GRN using ChIP-qPCR.**

Determination of the direct regulatory relationship among the TFs in second layer and the structural genes in the bottom layer. Three independent replicates were performed, and the error bars indicate the standard error. * Indicates a significant enrichment (P<0.05) compared with control (anti-HA).

**Figure S5**

**(a)**

**(b)**

**Figure S5 Determination of transgenic strains.**

Determination of the expression of *BpMADS11* and *BpNAC090* in transgenic lines (OE). (a) DNA of *OEBpMADS11* line is detected using PCR, and the relative expression of *BpMADS11* in OE lines was divided by that in WT plants. (b) PCR and RT-PCR identification of the *BpNAC090* overexpressing birch lines. The ratios were log2 transformed. And each sample contains at least 3 plantlets. The error bar represents the standard deviation (STD) of the three biological repetition. Asterisks indicate significant differences between transgenic strains and WT (P < 0.05).
