# Supplementary material for: Hierarchical transcription factor and regulatory network for drought response in Betula platyphylla
Source: Hortic Res. 2022 Feb 19;9:uhac040. doi: 10.1093/hr/uhac040 (PMC9070641; doi:10.1093/hr/uhac040)
Supplement: Web_Material_uhac040 [file web_material_uhac040.zip › Table S2 The enriched GO terms under drought stress conditions that were used to construct the gene regulatory network.docx]

**Table 2. The enriched GO terms under drought stress conditions that were used to construct the gene regulatory network.**

| GO terms | Fold Enrichment | FDR |
| --- | --- | --- |
| Jasmonic acid biosynthetic process | 9.0 | 8.95E-05 |
| Jasmonic acid metabolic process | 10.35 | 2.46E-07 |
| Water transport | 8.28 | 1.25E-03 |
| Response to oxidative stress | 2.69 | 2.27E-08 |
| Response to jasmonic acid | 3.73 | 1.07E-08 |
| Regulation of stomatal movement | 3.08 | 4.77E-02 |
| Response to water deprivation | 3.08 | 9.08E-09 |
| Response to abscisic acid | 2.49 | 2.91E-08 |
| Response to ethylene | 2.37 | 7.17E-04 |
| Response to osmotic stress | 2.55 | 4.89E-10 |
| Transmembrane transport of small molecules | 2.02 | 4.05E-02 |
| oxidoreductase | 2.62 | 1.65E-18 |
| dehydratase | 2.93 | 3.79E-03 |
| dehydrogenase | 2.36 | 1.20E-04 |
| reductase | 2.18 | 8.44E-03 |
